# Fyn Kinase: A Potential Target in Glucolipid Metabolism and Diabetes Mellitus

**DOI:** 10.3390/cimb47080623

**Published:** 2025-08-05

**Authors:** Ruifeng Xiao, Cong Shen, Wen Shen, Xunan Wu, Xia Deng, Jue Jia, Guoyue Yuan

**Affiliations:** Department of Endocrinology and Metabolism, Affiliated Hospital of Jiangsu University, Institute of Endocrine and Metabolic Diseases, Jiangsu University, Zhenjiang 212000, China; xiaoruifenga@163.com (R.X.);

**Keywords:** glucolipid metabolism, diabetes mellitus, Fyn kinase, diabetic complications, insulin, inflammation

## Abstract

Fyn is widely involved in diverse cellular physiological processes, including cell growth and survival, and has been implicated in the regulation of energy metabolism and the pathogenesis of diabetes mellitus through multiple pathways. Fyn plays a role in increasing fat accumulation and promoting insulin resistance, and it also contributes to the development of diabetic complications such as diabetic kidney disease and diabetic retinopathy. The primary mechanism by which Fyn modulates lipid metabolism is that it inhibits AMP-activated protein kinase (AMPK). Additionally, it affects energy homeostasis through regulating specific signal pathways affecting lipid metabolism including pathways related to CD36, through enhancement of adipocyte differentiation, and through modulating insulin signal transduction. Inflammatory stress is one of the fundamental mechanisms in diabetes mellitus and its complications. Fyn also plays a role in inflammatory stress-related signaling cascades such as the Akt/GSK-3β/Fyn/Nrf2 pathway, exacerbating inflammation in diabetes mellitus. Therefore, Fyn emerges as a promising therapeutic target for regulating glucolipid metabolism and alleviating type 2 diabetes mellitus. This review synthesizes research on the role of Fyn in the regulation of energy metabolism and the development of diabetes mellitus, while exploring its specific regulatory mechanisms.

## 1. Introduction

Type 2 diabetes mellitus (T2DM) is a progressive metabolic disease characterized by peripheral insulin resistance and is strongly associated with obesity and hyperlipidemia. T2DM is associated with a range of serious complications, such as diabetic nephropathy and coronary artery disease, which significantly impair the life quality of large populations worldwide [1,2]. Recent epidemiological data reveal that approximately 589 million adults worldwide are affected by diabetes in 2025, with projections indicating a continuous rise [3]. The pathogenesis of T2DM involves intricate signaling pathways, including the impairment of insulin receptor substrates (IRSs)/phosphatidylinositol-3-kinase (PI3K)/Akt/glucose transporter 4 (GLUT4) signaling, which leads to reduced GLUT4 translocation; dysfunction of the AMP-activated protein kinase (AMPK) pathway, which disrupts glucose and lipid metabolism; and activation of inflammatory signaling pathways [2,4]. Recently, increasing attention has been directed toward various molecular regulators of these processes. Among these molecular regulators, Fyn, a non-receptor tyrosine kinase, has emerged as a modulator of specific glucolipid metabolism signaling pathways such as AMPK pathways, and several inflammatory signaling pathways, thus being implicated in T2DM pathogenesis.

Fyn, one of crucial members of Src-family kinases (SFKs), catalyzes the phosphorylation of tyrosine residues on specific target proteins [5,6]. It contains four Src homology (SH) domains—the SH1, SH2, SH3, and SH4 domains, where SH1 is the catalytic domain and SH2 and SH3 are regulatory domains—and it is activated by phosphorylation through conformation changes [7,8]. There are three splice variants of Fyn in humans: Fyn*T*, Fyn*B*, and Fyn*Δ7.* These isoforms differ in a short peptide segment located between the SH1 and SH2 domains, with the Fyn*T* isoform appearing to exhibit higher catalytic activity [9]. Fyn is widely expressed across diverse types of cells and plays an essential role in numerous biological processes, including regulation of cell growth and survival, cell adhesion, integrin-mediated signaling, cytoskeletal remodeling, cell motility, immune response, axon guidance, and synaptic function [10,11,12,13]. Beyond these functions, growing evidence has indicated that Fyn is involved in glucolipid metabolism regulation, as well as in the pathogenesis and progression of diabetes mellitus and its complications [14,15,16,17,18,19,20,21,22,23,24,25,26,27,28].

## 2. Effects of Fyn on Glucolipid Metabolism

Fyn knockout (KO) mice or mice pharmacologically inhibited for Fyn exhibited significant reductions in fasting glucose, insulin, triglycerides, and free fatty acids (FFAs) in plasma, as well as reduced lipid accumulation in liver and muscle cells, and improved insulin sensitivity in peripheral tissues such as adipose tissue and skeletal muscle [14,15]. These metabolic benefits were also observed under high-fat diet (HFD) conditions: reduced fasting blood glucose, lipids, insulin, and FFAs and improved glucose tolerance [16]. Fyn also exhibited similar effects on lipid accumulation in an alcohol-induced fatty liver model: Fyn-deficient mice showed reduced liver steatosis [29].

FynKO mice exhibited decreased overall adipose mass due to a reduction in adipocyte cell size, and a shift in adipose distribution from visceral to subcutaneous depots [14,15,16]. Notably, while Fyn-deficient mice exhibited reduced body weight compared to wild-type mice under a chow diet [14], they were unable to prevent weight gain induced by an HFD [16]. This observation may be attributed to the following: the phenomenon that Fyn mainly influences the oxygenolysis of fatty acids (FAs) during fasting with a relatively minor impact during the fed state [14], and its facilitation of hepatic de novo gluconeogenesis during fasting [30].

Interestingly, the modulatory effects of Fyn on lipid homeostasis appear to be organ/tissue context-dependent: while Fyn promotes lipid accumulation in metabolic tissues like liver, adipose tissue, and skeletal muscle, it suppresses adipocyte formation in mouse long bone marrow mesenchymal stem cells [31].

## 3. Role of Fyn in the Development of Diabetic Complications

Consistent with the effects of Fyn on glucolipid metabolism, it also contributes to the development of diabetic complications such as diabetic kidney disease (DKD). The mechanisms through which Fyn contributes to diabetic microangiopathy, including DKD and diabetic retinopathy, have received more attention [18,19,20,21,22,23].

Fyn plays a role in numerous signaling pathways associated with renal injury in the context of DKD, affecting renal tubule cells, podocytes, and mesangial cells. In renal tubule cells, high glucose exposure activates Fyn, causing translocation of the downstream effector mechanistic target of rapamycin (mTOR) to the lysosomal membrane and its downstream signaling, which culminate in endoplasmic reticulum stress and inflammatory damage [32]. Additionally, Fyn phosphorylates transglutaminase 2 in diabetic renal tubular epithelial cells, thereby inhibiting autophagy, a crucial function for maintaining cellular homeostasis [18,33]. In podocytes, a high-glucose environment activates Fyn, which subsequently activates the Rho-associated coiled-coil-forming protein kinase and its downstream cascade signals, increasing motility of podocytes [19]. Moreover, Fyn has also been identified as a putative mediator of the function of Src homology phosphatase 2 (Shp2), a protein associated with podocyte migration induced by high glucose [20]. In addition, nuclear receptor coactivator 3 (NCOA3) downregulates Fyn transcription in a peroxisome proliferator-activated receptor γ (PPARγ)-dependent manner, leading to upregulation of autophagy flux in podocytes [21]. In mesangial cells, Fyn tyrosine-phosphorylates c-Cbl and triggers ubiquitination and degradation of sirt1, thereby negatively regulating the Sirt1/Foxo3a antioxidant pathway, and exacerbating diabetic renal fibrosis [34]. Consistent with these findings, several bioinformatic analyses have highlighted that Fyn is one of the key genes in the signaling pathways associated with the development of DKD [35,36,37].

In addition to its role in DKD, Fyn is also implicated in the development of diabetic retinopathy. Human retinal vascular endothelial (hRVE) cells are target cells for diabetes-induced vascular damage [38,39]. Fyn contributes to retinal vascular dysfunction by modulating inflammatory responses and endothelial permeability. Firstly, the reduction of Fyn and other SFK members within caveolae/lipid raft microdomains of hVRE cells reduced tumor necrosis factor-α (TNF-α)-induced expression of vascular cell adhesion molecule-1 (VCAM-1), and was linked to the anti-inflammatory effects of docosahexaenoic acid (DHA) in hRVE cells. This suggests that Fyn plays a critical role in the inflammatory responses in diabetic retinopathy pathogenesis [22]. Secondly, Fyn is involved in the role of tubedown in proliferative diabetic retinopathy (PDR). Tubedown may mediate the inhibition of increased retinal vascular endothelial cell permeability in PDR through negatively regulating the permeability of these cells to albumin. This effect is mediated by the inhibition of cortical protein phosphorylation and subsequent inhibition of SFK, including Fyn [23]. These findings suggest that Fyn may have an adverse effect on the progression of diabetic retinopathy.

In addition to diabetic microangiopathy, emerging evidence indicates that Fyn also plays a role in other diabetic complications, such as diabetic cardiomyopathy and diabetic neuropathy.

In diabetic cardiomyopathy, Fyn exacerbates cardiac dysfunction and promotes inflammatory responses and myocardial fibrosis in cardiomyocytes via the NORAD/miR-125a-3p/Fyn network. The long non-coding RNA NORAD adsorbs miR-125a-3p and enhances the pathological effects mediated by Fyn in diabetic cardiomyopathy [24]. In diabetic neuropathy, Fyn mediates phosphorylation of the NR2B subunit of the N-methyl-D-aspartate (NMDA) receptor, contributing to neuronal dysfunction, which has been experimentally confirmed in the spinal cords and hippocampi of mice [40,41].

## 4. Role of Fyn in Signaling Pathways Associated with Glucolipid Metabolism

### 4.1. Mechanisms of Fyn in Lipid Metabolism

#### 4.1.1. Role of Fyn in Signaling Pathways Directly Affecting Lipid Metabolism

The metabolic improvements observed in FynKO mice or mice treated with an inhibitor of Fyn, such as reduced FFAs in plasma and lipid accumulation in cells, are mainly attributed to increased oxidative decomposition and decreased FA synthesis in adipose tissue and skeletal muscle [14,42]. The key mechanism underlying these changes in lipid metabolism is that Fyn directly or indirectly inhibits the activation of AMPK in adipose tissue and skeletal muscle [17].

AMPK is a key metabolic regulator that inhibits lipid accumulation. AMPK suppresses the conversion of acetyl-CoA to malonyl-CoA by the inhibitory phosphorylation of acetyl-CoA carboxylase (ACC). Low levels of malonyl-CoA limit FA synthesis, and promote FA oxidation by enhancing the activity of carnitine palmitoyl transferase 1 (CPT-1), the rate-limiting enzyme in FA oxidation. Fyn exerts its inhibitory effect on AMPK through specific molecular interactions [14,15,17].

Fyn inhibits AMPK activity by binding its SH3 domain to the proline-rich region of liver kinase B1 (LKB1), a major AMPK kinase, and catalyzes its inhibitory phosphorylation at the Y261 and Y365 sites [17]. This modification increases the nuclear localization of LKB1, thereby inhibiting AMPK in adipocytes and skeletal muscle cells. Consequently, FA oxidation is suppressed, and lipid synthesis is promoted [15]. Interestingly, the impact of Fyn on AMPK activity may display cell-type specificity. One study suggests that the Src kinase inhibitor SU6656 directly inhibits AMPK kinase instead of this signaling pathway in human embryonic kidney 293 cells and mouse embryo fibroblasts (MEFs), independently of the aforementioned regulatory role of Fyn [43].

Beyond the AMPK pathway, Fyn also affects lipid metabolism through the signal transducer and activator of transcription 5a (STAT5a) pathway and Thy1-PPARγ pathway in adipocytes: Fyn activates STAT5a, a transcription factor in triggering lipogenesis, by phosphorylating phosphoinositide 3-kinase enhancer A (PIKE-A), which is a pathway of prolactin-induced lipogenesis [44]. Fyn also increases the transcriptional activity of the adipogenic transcription factor PPARγ, thereby promoting adipogenesis and contributing to obesity, which is inhibited by the cell membrane protein Thy1 [45].

#### 4.1.2. Role of Fyn in Mechanisms of CD36 Affecting Lipid Metabolism

CD36 is a membrane glycoprotein involved in long-chain FA uptake, lipid accumulation, and metabolic dysfunction in the presence of excess fat supply [46]. Fyn plays a significant role in lipid metabolism through its interaction with CD36.

Fyn modulates CD36-mediated lipid metabolism primarily by inhibiting lipolysis in response to insulin. This process is mediated through the following mechanisms: Firstly, as previously stated, Fyn facilitates the translocation of LKB1 to the nucleus, thus reducing the phosphorylation of AMPK, which ultimately reduces the catabolism of FA. CD36 dissociates Fyn from the ternary complex composed of Fyn, LKB1, and AMPK under the stimulation of extracellular FAs and promotes lipid accumulation via inhibiting the AMPK pathway in skeletal muscle and the liver [47,48,49,50]. Secondly, Fyn is involved in the interaction of ubiquitinated CD36 and IRS1 and prevents IRS1 degradation by preventing its interaction with cullin 7, thereby delaying but sustaining insulin signaling in muscle [51]. In addition, Fyn is crucial in CD36-mediated enhancement of lipid synthesis, digestion, and absorption [52,53]. Moreover, Fyn indirectly promotes fat intake by enhancing perception of fat taste after FA binding to CD36 in taste cells [54,55]. In summary, Fyn contributes to CD36-mediated lipid accumulation.

Consistent with Fyn-mediated regulation of lipid metabolism through CD36, emerging evidence has suggested that Fyn is probably implicated in various pathological states related to dysregulated lipid homeostasis mediated by CD36. Fyn contributes to the progression of the formation of atherosclerotic plaque mediated by CD36, including promoting oxidized low-density lipoprotein (OxLDL)-induced prethrombotic states [56,57] and increasing the formation of foam cells [58]. In contrast, some studies have shown that Fyn is involved in the process in which OxLDL binds to CD36 and inhibits atherosclerosis in the early stages [59]. This duality may be attributed to the multifaceted role of the interaction between OxLDL and CD36 during different stages of atherogenesis. Additionally, Fyn–CD36 interactions contribute to metabolic inflammation. In preadipocytes, Fyn phosphorylates inositol (1,4,5)-trisphosphate receptor 1(IP3R1), thereby mediating lysosomal calcium ion overload in response to upregulation of CD36, which promotes inflammation in adipose tissue [60]; in microglia, Fyn mediates CD36-induced mitochondrial dysfunction and activation of the Nod-like receptor protein 3 (NLRP3) inflammasome in response to α-synuclein, though the relevance of this mechanism to hyperlipidemia-associated inflammation requires further investigation [61]. Collectively, the above discoveries suggest that Fyn is involved in pathological states associated with CD36-driven aberrant lipid metabolism.

#### 4.1.3. Role of Fyn in Adipocyte Differentiation

In addition to the mechanisms regulating lipid metabolism mentioned above, Fyn is involved in signaling pathways relevant to adipocyte differentiation. Fyn exhibits distinct activity, expression levels, and subcellular locations at different stages of adipocyte differentiation. Specifically, it has been shown that Src-family kinase inhibitors prevent adipocyte differentiation [42,62], while Fyn expression is upregulated during the transition of preadipocytes to mature adipocytes in both humans and mice [44,62]. Fyn differentially affects the tyrosine phosphorylation of caveolin stimulated by insulin in preadipocytes and adipocytes [63]. Moreover, flotillin-1 relocates from late endosomal/lysosomal vesicles to plasma membrane lipid rafts during the differentiation process from preadipocyte to adipocyte, and Fyn also exhibits a similar differentiation-dependent lipid raft distribution [64]. These findings collectively suggest that Fyn may serve as an important intermediate protein in adipocyte differentiation.

Regarding the possible mechanism, firstly, Fyn activates STAT5a by enhancing the interaction between PIKE-A and STAT5a, which may be one of the links enhancing STAT5a-mediated adipocyte differentiation [44]. Secondly, the interaction between metastasis suppressor 1 (MTSS1) and protein tyrosine phosphatase receptor-δ (PTPRD) leads to decreased phosphorylation of Fyn at Tyr530 and increased phosphorylation at Tyr419, which activates Fyn and drives the differentiation of mesenchymal progenitor cells into adipocytes [65]. 

In summary, Fyn appears to positively regulate adipocyte differentiation, thereby promoting lipid accumulation and corresponding metabolic characteristics.

### 4.2. Role of Fyn in Insulin Signaling

#### 4.2.1. Direct Interactions Between Fyn and Insulin Signaling Components

Fyn is potentially involved in insulin signaling through direct interactions with key components of the pathways, as suggested by many in vitro studies. Firstly, Fyn has been shown to bind to IRS1 and IRS5/DOK4 (downstream of kinase 4) in response to insulin stimulation and is involved in their tyrosine phosphorylation, which indicates a direct role in insulin signaling pathways [66,67]. Secondly, Fyn exhibits an inhibitory effect on insulin signaling by facilitating the function of growth factor receptor-bound protein 10 (Grb10), a negative regulator of insulin signaling [68,69]. Thirdly, Fyn may also regulate insulin signaling pathways through associating with c-Cbl and catalyzing its tyrosine phosphorylation in adipocytes upon insulin stimulation [70,71]. Interestingly, it has been reported that c-Cbl may promote the degradation of Fyn by enhancing its ubiquitination [72]. Further studies are required to confirm whether c-Cbl also negatively regulates the catalytic activity of Fyn in adipocytes through this mechanism.

#### 4.2.2. Role of Fyn in Insulin Signaling Through Lipid Raft

It is noteworthy that Fyn regulates insulin signaling through lipid raft-dependent mechanisms. Caveolae, as a major type of lipid raft, play a crucial role in regulating insulin secretion and insulin signaling because insulin receptors (IRs) are predominantly localized in caveolae on the plasma membrane [73,74]. Fyn is localized in caveolae and is essential for their structural integrity and functions including those related to insulin signal transduction [75,76,77,78,79]. This suggests that Fyn may be involved in the mechanism of insulin resistance.

One key mechanism through which Fyn regulates insulin resistance is FA modification. This may represent one of the mechanisms by which saturated fatty acids (SFAs) affect insulin resistance. Studies have demonstrated that targeting of Fyn to lipid rafts in the plasma membrane requires modifications such as palmitoylation and myristoylation after the synthesis of Fyn in the cytoplasm [80,81,82]; similarly, the intake of unsaturated fatty acids (UFAs) may modulate insulin signal transduction through this Fyn-dependent mechanism. Fyn undergoes S-acylation with several UFAs and reduces its binding to the lipid raft, resulting in inhibition of related downstream signal transduction [83]. 

Another potential pattern of Fyn’s regulation of caveolae in insulin signaling is insulin-like growth factor (IGF)-receptor crosstalk. Studies have found that IGF-receptor activation in caveolae recruits Fyn to the caveola region, where it catalyzes the phosphorylation of Shc and activates the mitogen-activated protein kinase (MAPK) pathway [84].

Caveolin-1 (CAV1), an indispensable component of caveolae, is required for the regulation of Fyn in insulin signaling. CAV1 facilitates the concentration of insulin receptors and downstream effector proteins within caveolae, thereby playing an important role in insulin resistance and the pathogenesis of T2DM [85]. Fyn regulates insulin signaling through CAV1 via the following key mechanisms: Firstly, Fyn is associated with phosphorylation of CAV1. Fyn catalyzes the tyrosine phosphorylation of caveolin-associated protein pp29 and protein pp36 [86], so it can be inferred that Fyn plays a role in insulin-induced tyrosine phosphorylation of caveolin [87] in adipocytes through this mechanism [63]. Consistent with these findings, studies have discovered that the synergistic effect of activated Fyn and Abl is required for CAV1 phosphorylation [88]. In addition, the interaction between Fyn and caveolin residues 82-101 negatively regulates the auto-activation of Fyn, indicating a feedback mechanism [76]. Secondly, Fyn also plays a role in CAV1-mediated GLUT4 translocation in response to insulin. CAV1 mediates insulin-induced GLUT4 translocation through its binding to IRs [85]. Insulin-mediated GLUT4 translocation associated with caveolae rosettes in adipocytes is mediated by cyclin-dependent kinase-5 (CDK5)-dependent phosphorylation of the Rho family GTPase TC10 (α), while this phosphorylation is catalyzed by insulin-stimulated activated Fyn [89].

**Figure 1:**
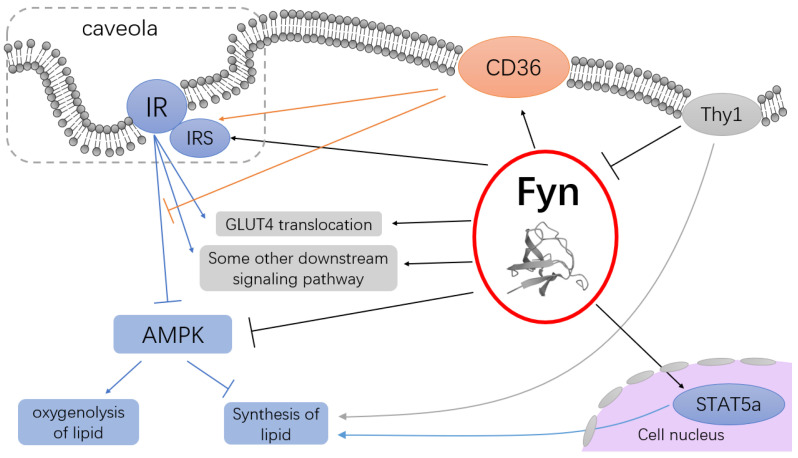
Several signaling pathways of glucolipid metabolism modulated by Fyn. Fyn plays a role in the inhibition of specific downstream signaling pathways following insulin receptor stimulation through CD36-mediated inhibition of AMPK activation, among other pathways. These interactions lead to metabolic alterations such as increased lipogenesis and decreased lipolysis. Additionally, Fyn regulates lipid metabolism through its effects on molecules such as STAT5a and Thy1. Arrow notation: common arrow—promotion; T-shaped arrow—inhibition (the structure of the protein was obtained from UniProt).

In summary, Fyn plays a regulatory role in glucolipid metabolism, primarily by modulating lipid metabolism through the inhibition of the AMPK signaling pathway. Additionally, Fyn participates in the modulation of insulin signaling pathways (Figure 1).

### 4.3. Role of Fyn in the State of Inflammation Associated with Hyperglycemia and Hyperlipidemia

Under the condition of elevated glucose and lipid levels, inflammatory stress is intensified [90], with Fyn involved in the process. This effect is supported by the finding that the inflammation of adipose tissue induced by high-fat feeding in FynKO mice was alleviated [16]. Fyn mainly participates in this inflammatory response through two key signaling pathways: the Akt/glycogen synthase kinase 3β/Fyn/nuclear factor erythroid 2-related factor 2 (Akt/GSK3β/Fyn/Nrf2) signaling pathway and G-protein-coupled receptor family C group 5 member B (GPRC5B)/Fyn signaling pathway [25,26,27,28].

#### 4.3.1. Akt/GSK3β/Fyn/Nrf2 Signaling Pathway

Nrf2, a transcriptional regulator of antioxidant responses, activates the expression of antioxidant genes such as heme oxygenase-1 (HO-1) and NAD(P)H quinone dehydrogenase 1 (NQO1) through binding to antioxidant response elements (AREs). Fyn negatively regulates Nrf2 by promoting its phosphorylation, nuclear export, ubiquitination, and subsequent degradation [91]. This regulatory axis is particularly relevant in diabetes mellitus, where oxidative stress induces diminished Akt activation, leading to reduced inhibition of GSK3β. In turn, active GSK3β activates Fyn, further suppressing Nrf2-mediated antioxidant defenses [25,26,27].

The antioxidant effect of Nrf2 ameliorates insulin resistance and promotes energy consumption by mechanisms such as protecting pancreatic β-cells from oxidative stress [92,93]. This effect of Nrf2 also alleviates diabetic complications like DKD. It attenuates renal injury through protective mechanisms including reducing hyperglycemia-induced renal integrative glomerular barrier complex dysfunction [94]. Additionally, in macrophages, SFAs promote Fyn nuclear translocation, where it inhibits Nrf2 signaling, further aggravating oxidative stress in high-fat metabolic environments [95].

Some antioxidant substances, such as liraglutide, Baicalin, and mango ginger extract, have been proved to alleviate oxidative stress of diabetes through the Akt/GSK3β/Fyn/Nrf2 signaling pathway [96,97,98]. Therapeutic strategies targeting the Akt/GSK3β/Fyn/Nrf2 pathway have shown promise in mitigating diabetic oxidative stress (Table 1).

#### 4.3.2. GPRC5B Signaling Pathway

GPRC5B, an obesity-related membrane protein implicated in insulin resistance, may contribute to the metabolic disturbances characteristic of type 2 diabetes mellitus (T2DM), including increased adiposity, impaired insulin secretion, and glucose intolerance [28,107]. Emerging evidence indicates that Fyn serves as a key mediator of GPRC5B’s metabolic effects.

The interaction between GPRC5B and Fyn triggers local amplification of Fyn kinase activity, subsequently activating the inhibitor of the κB (IκB) kinase ε (IKKε)–nuclear factor κB (NFκB) signaling axis. This activation initiates an inflammatory positive feedback loop that promotes adipose tissue inflammation and contributes to diet-induced obesity and insulin resistance [28]. Similarly, in vascular cells, elevated glucose levels and inflammatory cytokines upregulate GPRC5B, leading to Fyn-mediated phosphorylation of extracellular signal-regulated kinase 1/2 (ERK1/2) and consequent activation of the NFκB pathway [108]. This mechanism represents a crucial link between hyperglycemia and the development of vascular inflammation and atherosclerosis.

Furthermore, GPRC5B facilitates Fyn-dependent phosphorylation of sphingomyelin synthase 2 (SMS2), which activates the diacylglycerol/protein kinase C/c-Jun N-terminal kinase (DAG/PKC/JNK) signaling cascade, a key pathway in the pathogenesis of insulin resistance [109]. These findings collectively demonstrate that GPRC5B exerts its metabolic effects through Fyn.

#### 4.3.3. Insulin-Mediated Mast Cell Response

**Figure 2:**
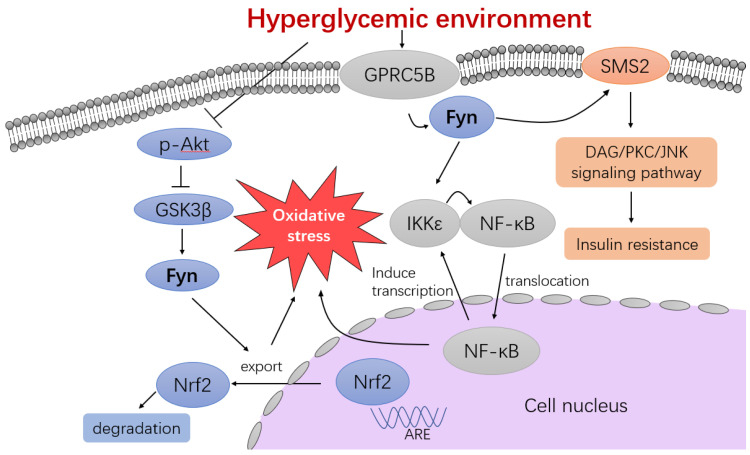
Fyn is involved in signaling pathways exacerbating cellular oxidative stress induced by hyperglycemia. Prolonged hyperglycemia inhibits Akt phosphorylation, leading to reduced suppression of GSK3β. This promotes Fyn activation and results in the nuclear exportation and degradation of Nrf2, an antioxidant factor. Additionally, in a hyperglycemic environment, Fyn activated by GPRC5B initiates the IKKε-NF-κB positive feedback loop and the SMS2 signaling pathway, contributing to oxidative stress and insulin resistance, respectively.

**Figure 3:**
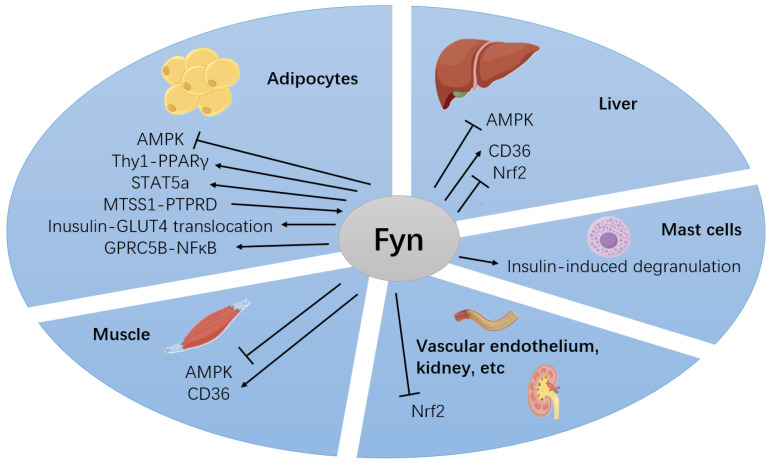
Fyn differentially regulates glucolipid metabolism-associated signaling pathways across different tissues. In adipose tissue, Fyn modulates glucolipid metabolism through multiple pathways including AMPK signaling, Thy1-PPARγ axis, STAT5a signaling, and insulin signaling cascades. It also contributes to hyperglycemia-induced inflammation via the GPRC5B-NFκB pathway. In skeletal muscle and the liver, Fyn regulates metabolic processes mainly through CD36-mediated inhibition of AMPK activation. Additionally, in the liver, Fyn promotes hyperglycemia/hyperlipidemia-induced inflammatory states by suppressing Nrf2. Furthermore, in the vascular endothelium and kidney, Fyn exacerbates glucolipid-induced oxidative stress mainly through Nrf2 inhibition, thus being involved in diabetic complications. Moreover, Fyn also enhances diabetic inflammation via insulin-mediated mast cell degranulation (by Figdraw).

In addition to the Akt/GSK-3β/Fyn/Nrf2 and GPRC5B/Fyn signaling pathways, Fyn is also implicated in insulin-mediated mast cell response. Increasing evidence indicates that mast cells are involved in the development of insulin resistance and T2DM [110,111]. Insulin regulates antigen-mediated responses of bone marrow-derived mouse mast cells by enhancing IgE-mediated mast cell degranulation, with Fyn implicated in this downstream signaling pathway of insulin [112], which may be one of the mechanisms underlying the pathogenesis of diabetes mellitus (Table 2, Figure 2 and Figure 3).

## 5. Conclusions

Fyn is involved in the regulation of glucolipid metabolism and the pathogenesis of diabetes mellitus and its complications through diverse mechanisms in various tissues (Figure 3, Table 2). Primarily, Fyn suppresses AMPK activity, thereby inhibiting FA oxidation and promoting FA synthesis, which contributes to increased adiposity and reduced insulin sensitivity. Beyond this pathway, Fyn affects insulin signaling pathways and energy metabolism through diverse signaling pathways, including regulation of lipid metabolism via CD36-mediated FA uptake and via adipocyte differentiation; insulin signaling modulation through lipid raft-dependent pathways; and involvement of inflammatory responses, such as the Akt/GSK-3β/Fyn/Nrf2 and GPRC5B/Fyn pathways. Notably, a variety of drugs alleviate diabetes mellitus through the Akt/GSK-3β/Fyn/Nrf2 signaling pathway. Therefore, targeted inhibition of Fyn represents a potential novel strategy for reduction of adipose accumulation and alleviation of obesity, as well as a promising approach for treating type 2 diabetes mellitus and its complications.

Despite these advances, significant gaps remain regarding the specific mechanisms of Fyn in energy metabolism and diabetes mellitus. Since current research tools of Fyn, such as Fyn knockout animal models and Fyn inhibitors, target all isoforms of Fyn without distinction, future studies should aim to elucidate the distinct role of each Fyn isoform in the regulation of glucolipid metabolism across different cell types, like adipocytes. Further research is needed to explore uncertainties, such as the precise mechanisms by which Fyn impacts body weight under different dietary conditions, the effects of Fyn on the insulin signaling pathway in vivo, whether Fyn also inhibits AMPK through CD36 in adipocytes, and whether the mechanism by which Fyn regulates insulin signaling pathways through c-Cbl in skeletal muscle cells and hepatocytes is the same as in adipocytes, among others. Although FynKO animal models have demonstrated metabolic benefits, the development of pharmacological Fyn inhibitors faces translational challenges. Given that Fyn is implicated in a wide range of cellular physiological activities, the design of adipocyte-selective Fyn inhibitors that preserve its essential biological functions will enhance clinical applicability, which remains a worthy research direction. Unraveling the enigmas surrounding the role of Fyn in metabolism, including the answers to these questions, will likely uncover novel therapeutic strategies for the treatment of metabolic disease.

## Figures and Tables

**Table 1:** Drugs (or receptors) playing a role in diabetes mellitus via the Akt/GSK-3β/Fyn/Nrf2 signal pathway.

Drug (or Receptor)	Species	The Effect of the Drug (or Receptor) via the Akt/GSK-3β/Fyn/Nrf2 Signal Pathway	Intermediate Links Between the Drug (or Receptor) And the Akt/GSK-3β/Fyn/Nrf2 Pathway	The Main Action of the Drug (or Receptor)	References
Liraglutide	Mouse	Ameliorates the impairment of endothelial progenitor cell functions under diabetic condition	-	Treats T2DM	[96]
Baicalin	Mouse and human	Ameliorates the oxidative stress state in vascular endothelial cells	-	Antibacterial, anti-inflammatory, cholesterol-lowering, antithrombotic, etc.	[97]
Mango ginger (*Curcuma amada Roxb*.)	Rat	Antioxidant, alleviates insulin resistance	-	Anti-inflammatory, antioxidant, antibacterial, anti-cancer, antihyperglycemic	[98]
CXC chemokine receptor 7	Mouse	Improves the angiogenic capability of endothelial progenitor cells in T2DM	-	Involved in cell proliferation	[99]
Fenofibrate	Mouse	Ameliorates DKD	Upregulates fibroblast growth factor 21	Reduces blood lipid	[100,101]
Sulforaphane	Rat and mouse	Ameliorates DKD	Through AMPK-α2	Antioxidant, anti-cancer, etc.	[102,103]
Zinc	Mouse and human	Alleviates the liver impairment induced by diabetes mellitus, ameliorates DKD	-	Promotes growth and immune function, maintains cell membrane structure, etc.	[27,104]
Resveratrol	Mouse	Alleviates osteoblast dysfunction induced by hyperglycemia	-	Antioxidant, anti-cancer, cardiovascular-protective, etc.	[105]
Catalpol	Mouse	Ameliorates osteoblast dysfunction induced by hyperglycemia, promotes osseointegration of titanium implants	-	Anti-inflammatory, anti-cancer, hypoglycemic, etc.	[106]

**Table 2:** Fyn-mediated regulation of signal pathways associated with glucolipid metabolism across cell types.

Type of Cell	Signal Pathway	Effect
Adipocytes	Inhibits AMPK activation	Reduces the oxidation of FAs and promotes lipogenesis
	Participates in Thy1-PPARγ pathway	Promotes lipogenesis
	Activates STAT5a	Promotes lipogenesis and adipocyte differentiation
	Participates in MTSS1-PTPRD pathway	Promotes adipocyte differentiation
	Participates in insulin-induced GLUT4 translocation	Promotes glucose uptake
	Participates in GPRC5B-NFκB pathway	Promotes inflammation
Skeletal muscle cells	Inhibits AMPK activation through CD36	Reduces the oxidation of FAs and promotes lipid synthesis
	Prevents IRS1 degradation	Delays but sustains insulin signaling
Hepatocytes	Inhibits AMPK activation through CD36	Reduces the oxidation of FAs and promotes lipid synthesis
	Inhibits Nrf2	Promotes inflammation in diabetes mellitus
Mast cells	Enhances IgE-mediated mast cell degranulation	Promotes inflammation in diabetes mellitus
Macrophages	Inhibits Nrf2	Promotes inflammation in hyperlipidemia
Vascular endothelial cells	Inhibits Nrf2	Promotes inflammation in diabetes mellitus
Vascular smooth muscle cells	Participates in GPRC5B-NFκB pathway	Promotes inflammation in hyperglycemia
Renal tubule cells	Inhibits Nrf2; through mTOR pathway; through Transglutaminase2 pathway	Promotes inflammation in DKD
Podocytes	Activates Rho-associated coiled-coil-forming protein kinase pathway; participates in NCOA3 pathway	Promotes inflammation in DKD
Mesangial cells	Inhibits Nrf2; negatively regulates Sirt1/Foxo3a pathway	Promotes inflammation in DKD
Retinal vascular endothelial cells	Promotes TNF-α-VCAM-1 pathway; participates in role of tubedown	Promotes inflammation in diabetic retinopathy
Gustatory cells	Participates in CD36-mediated calcium ion influx	Increases perception of fat taste
Long bone marrow mesenchymal stem cells	Activates mTORC2	Inhibits adipocyte formation
